# *GlycomeDB *– integration of open-access carbohydrate structure databases

**DOI:** 10.1186/1471-2105-9-384

**Published:** 2008-09-19

**Authors:** René Ranzinger, Stephan Herget, Thomas Wetter, Claus-Wilhelm von der Lieth

**Affiliations:** 1 German Cancer Research Center (DKFZ), Core Facility: Molecular Structural Analysis, Im Neuenheimer Feld 280, D-69120, Heidelberg, Germany; 2 University of Heidelberg, Institute for Medical Biometry und Informatics, Im Neuenheimer Feld 305, D-69120, Heidelberg, Germany

## Abstract

**Background:**

Although carbohydrates are the third major class of biological macromolecules, after proteins and DNA, there is neither a comprehensive database for carbohydrate structures nor an established universal structure encoding scheme for computational purposes. Funding for further development of the Complex Carbohydrate Structure Database (CCSD or CarbBank) ceased in 1997, and since then several initiatives have developed independent databases with partially overlapping foci. For each database, different encoding schemes for residues and sequence topology were designed. Therefore, it is virtually impossible to obtain an overview of all deposited structures or to compare the contents of the various databases.

**Results:**

We have implemented procedures which download the structures contained in the seven major databases, e.g. GLYCOSCIENCES.de, the Consortium for Functional Glycomics (CFG), the Kyoto Encyclopedia of Genes and Genomes (KEGG) and the Bacterial Carbohydrate Structure Database (BCSDB). We have created a new database called *GlycomeDB*, containing all structures, their taxonomic annotations and references (IDs) for the original databases. More than 100000 datasets were imported, resulting in more than 33000 unique sequences now encoded in *GlycomeDB *using the universal format GlycoCT. Inconsistencies were found in all public databases, which were discussed and corrected in multiple feedback rounds with the responsible curators.

**Conclusion:**

*GlycomeDB *is a new, publicly available database for carbohydrate sequences with a unified, all-encompassing structure encoding format and NCBI taxonomic referencing. The database is updated weekly and can be downloaded free of charge. The JAVA application *GlycoUpdateDB *is also available for establishing and updating a local installation of *GlycomeDB*. With the advent of *GlycomeDB*, the distributed islands of knowledge in glycomics are now bridged to form a single resource.

## Background

A common problem for medieval European city-states was their autonomous regulations, currencies, weights and measures, which hampered trade exchange. An analogous lack of standardization is also a major obstacle in research projects using databases and bioinformatics services [1]. This problem is especially evident for carbohydrate databases, where sequence information is spread in incompatible formats over several unconnected databases. Here we report the results and the peculiarities of a data integration effort which aims to overcome the disadvantages inherent in the scattering of data in isolated carbohydrate databases.

The first publicly available carbohydrate structure database and the mother of all carbohydrate sequence databases is the Complex Carbohydrate Structure Database (CCSD), often called CarbBank in reference to the retrieval software used to access the data [2,3]. CarbBank was developed and maintained by the Complex Carbohydrate Research Center of the University of Georgia (USA). In the 1990s, it was the largest effort to collect glycan structures, mainly through retrospective manual extraction from the literature. However, funding for further development and maintenance of CarbBank was terminated in 1997, and the database has not been updated since then. Nevertheless, with about 50000 entries and more than 23000 different sequences, the CarbBank is still the largest repository of glycan data available.

After funding for CarbBank ceased, several other initiatives created new databases which imported subsets of CarbBank, e.g. GLYCOSCIENCES.de [4], the Bacterial Carbohydrate Structure Database (BCSDB) [5] and the glycan database of the Kyoto Encyclopedia of Genes and Genomes (KEGG) [6]. Each of these initiatives added new sequences, derived from retrospective literature analysis or new experimental evidence. Other databases for carbohydrate structures were created independently of CarbBank. Some of these were developed by commercial enterprises, i.e. GlycoSuite [7,8] and GlycoMinds [9], while others were created by scientific research groups or organizations, i.e. GlycoBase from the National Institute for Bioprocessing Research and Training (NIBRT) [10] (in the following listed as GlycoBase (Dublin)) and GlycoBase from the Université des Sciences et Technologies de Lille [11] (GlycoBase (Lille)). The Consortium for Functional Glycomics (CFG) [12,13] also established a glycan database using the commercial GlycoMinds data as a seed, to which they added new structures based on experimental evidence.

Almost all of the initiatives developed their own individual sequence encoding formats tailored to their specific needs, including the use of different naming conventions for the carbohydrate residues. Furthermore, the annotations (e.g. taxonomic information) are in different formats, and most of the databases offered no routines or strategy for automated data access, so that interested researchers were forced to crudely extract information directly from HTML pages ("screen scraping" [1]), for example. Consequently, each of the existing carbohydrate structure databases was an isolated island with a different "language", and comparison of the content was virtually impossible.

There were a few efforts to overcome this isolation problem: e.g. automated comparison of sequences implemented as a cross-link from the CFG to GLYCOSCIENCES.de (unpublished) or a cross-database search between GLYCOSCIENCES.de and BCSDB [14]. Some databases kept manually curated mappings of IDs from other databases (e.g. GlycoBase (Lille)) or the original references to CarbBank (e.g. KEGG). Generally, the cross-linking solutions implemented up to now have limitations since they are pairwise oriented only. Another problem of almost all recent databases is the lack of a tightly controlled and systematic vocabulary for the monosaccharides and their substituents. With thousands of different residues present in carbohydrate sequences, namespace inconsistencies can easily arise even within single databases.

Our work aims to integrate all available carbohydrate sequences into a single new database. Seven of the established carbohydrate structure databases follow an open-access strategy and are, thus, candidates for our data integration effort: BCSDB, CarbBank, CFG, GlycoBase (Dublin), GlycoBase (Lille), GLYCOSCIENCES.de and KEGG. We have implemented a JAVA software application called *GlycoUpdateDB*, which downloads the public databases listed above, reads their sequence notations, translates them to the GlycoCT encoding format [15] and a variant of Glyde [16], and stores the encoded sequences and corresponding IDs from the source databases in a new database called *GlycomeDB*. In addition, we have gathered and harmonized all of the taxonomic annotations available from the various databases. *GlycomeDB*, which is updated on a weekly basis, and *GlycoUpdateDB *are now publicly available and can be downloaded free of charge .

## Construction and content

**Figure 1:**
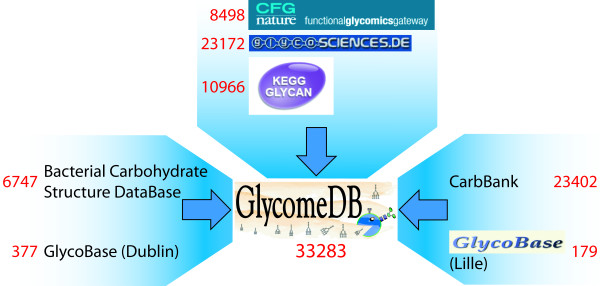
**The *GlycomeDB* concept**. *GlycomeDB *accesses all open-access carbohydrate databases and integrates the available structure and taxonomic information for all interpretable carbohydrate sequences using the unified structure encoding scheme GlycoCT. For each source database the total number of encoded structures currently available (August 2008) is shown in red.

Our goal was to integrate the heterogeneous resources of seven open-access carbohydrate databases into one central database called *GlycomeDB *(Figure 1). Approximately 100000 database records with 73341 sequences are accessible in the public domain. The following subsections describe the workflow implemented in the application called *GlycoUpdateDB*: data acquisition, integration of structural and taxonomic data and generation of the final database.

### Data acquisition

The first problem to solve was the accessibility of the structural data. The idea and first design studies for *GlycomeDB *date back to autumn 2005. At that time, only BCSDB and KEGG offered the direct download of their data, while CarbBank was available as a file on CD-ROM. The other database initiatives provided only HTML pages to access the data but no interfaces for automated data requests. To establish a stable and persistent mechanism for data export, we engaged in intensive communication with the database providers and finally convinced all of them to provide access to their structural and taxonomic data. Table 1 summarizes the download possibilities, which can now be executed without reliance on HTML pages for all initiatives.

**Table 1:** Mechanisms of data access from source databases

Database	Access Mode	URL	File Type
BCSDB	CGI		Text
CarbBank	HTTP		Text
CFG	CGI		CSV
GlycoBase (Dublin)	HTTP		CSV
GlycoBase (Lille)	CGI		XML
GLYCOSCIENCES.de	HTTP	and	CSV
KEGG	FTP		Text

The source databases used to obtain carbohydrate structures for *GlycomeDB *are listed together with their access mode, file type and URL. FTP and HTTP access is via normal file downloads; CGI means that a HTTP-GET or HTTP-POST request is made to a script residing on the remote server. File types are: Text = text with keywords, CSV = comma-separated values, XML = extensible markup language.

### Integration of carbohydrate sequences

**Figure 2:**
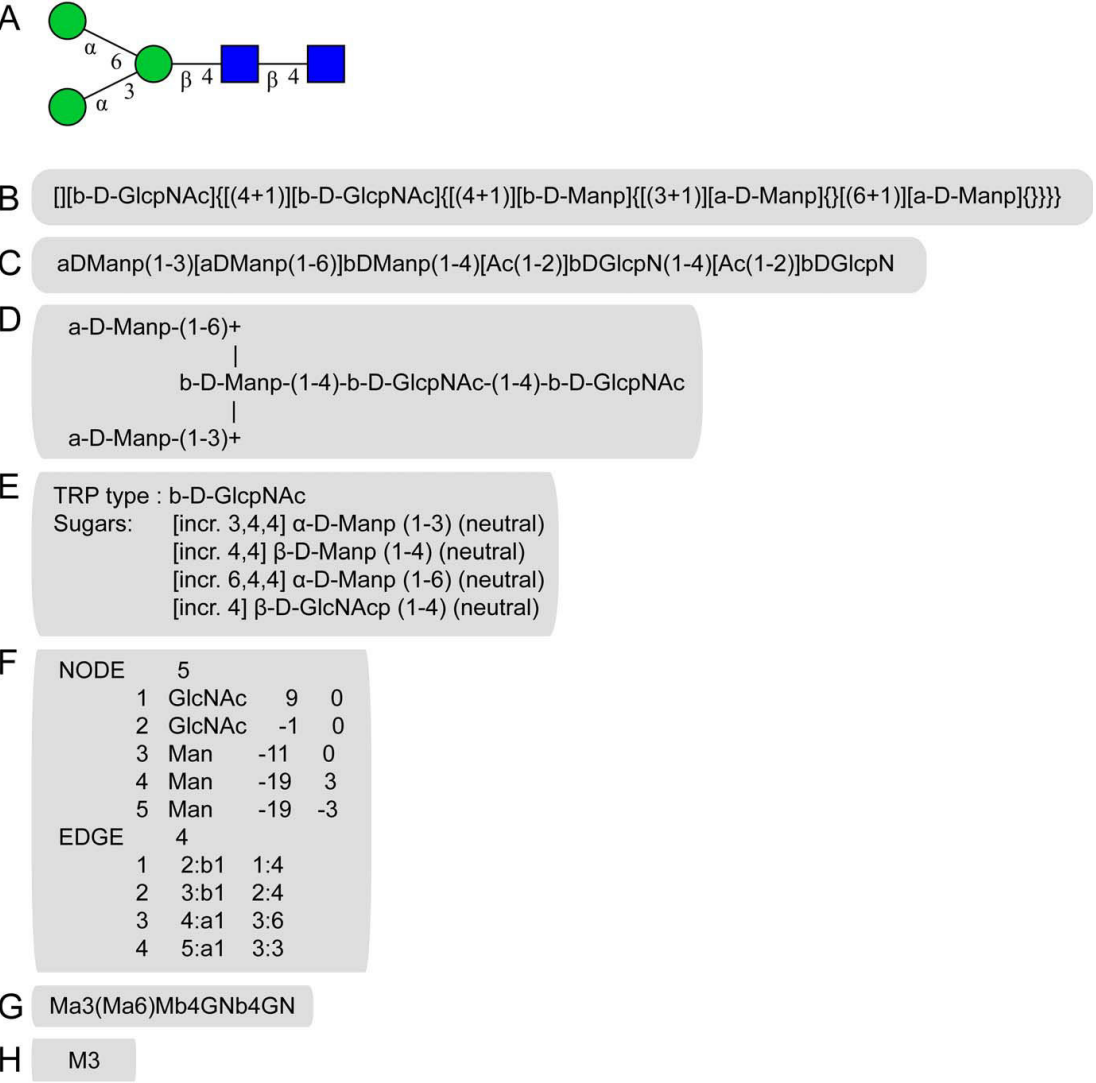
**Encoding schemes used in various carbohydrate sequence databases**. The *N*-glycan core structure has been chosen to illustrate the different encoding schemes used in the various databases for a given carbohydrate sequence. (**A) **Pictorial representation in CFG style with reducing end at the right. (**B) **LINUCS encoding used in GLYCOSCIENCES.de (reducing end at the left). **(C) **BCSDB encoding (reducing end at the right). **(D) **ASCII 2D graph as employed in CarbBank (reducing end at the right). **(E) **Notation used by GlycoBase (Lille). **(F) **KCF notation used by KEGG. **(G) **GlycoMinds encoding used in the CFG database (reducing end at the right). **(H) **Oxford notation used in GlycoBase (Dublin).

The biggest obstacle for data integration was the use of various sequence encoding formats by the different initiatives (Figure 2).

CarbBank employed 2D sketches of branched structures in ASCII format, closely resembling IUPAC recommendations [17]. GLYCOSCIENCES.de and BCSDB developed independently linear sequence encoding schemes which were able to create unique ASCII strings for each sequence [14,18]. KEGG was the first carbohydrate structure database to use a connection table approach (KCF) [19]. GlycoBase (Lille) follows a similar approach, storing the reducing end residue as an anchor point with all other residues specified in terms of relative position information [11]. The CFG database utilizes the LinearCode^® ^developed by Glycominds, the only format with a controlled namespace for monosaccharides and substituents [20]. All other encodings use free-text names for residues. The sequence format developed for GlycoBase (Dublin) is based on a motif encoding [10,21].

Since none of the existing solutions is truly capable of encompassing the whole problem space of carbohydrate sequences, we found it necessary to develop a new sequence format, called GlycoCT, which is a superset of the structural encoding capabilities inherent in all other formats developed so far [15]. We have implemented a translation library, which can read all of the carbohydrate sequence formats described above (parsing process) and translate them to GlycoCT.

To develop the parsing routines, we first carefully analyzed all existing formats and extracted their grammars, which are available in Extended Backus Naur Form (EBNF) [22] in Additional file 1. We then implemented parsers which can accept as input a sequence based on any of the documented notations and grammars. All parsers, except the one for the CarbBank notation, are implemented as recursive descent parsers [23]. During a second phase, the residue names must be translated to a harmonized format since most databases use free-text identifiers for the monosaccharides. For example, a monosaccharide can have different but similar text representations in the databases (e.g. α-D-mannose = aDMan, a-D-Manp or a-Man). Furthermore, trivial names for monosaccharides are commonly used in addition to systematic names: e.g. GLYCOSCIENCES.de uses both a-L-6-deoxy-Glc*p*N and a-L-Qui*p*N as synonyms for the same monosaccharide.

**Figure 3:**
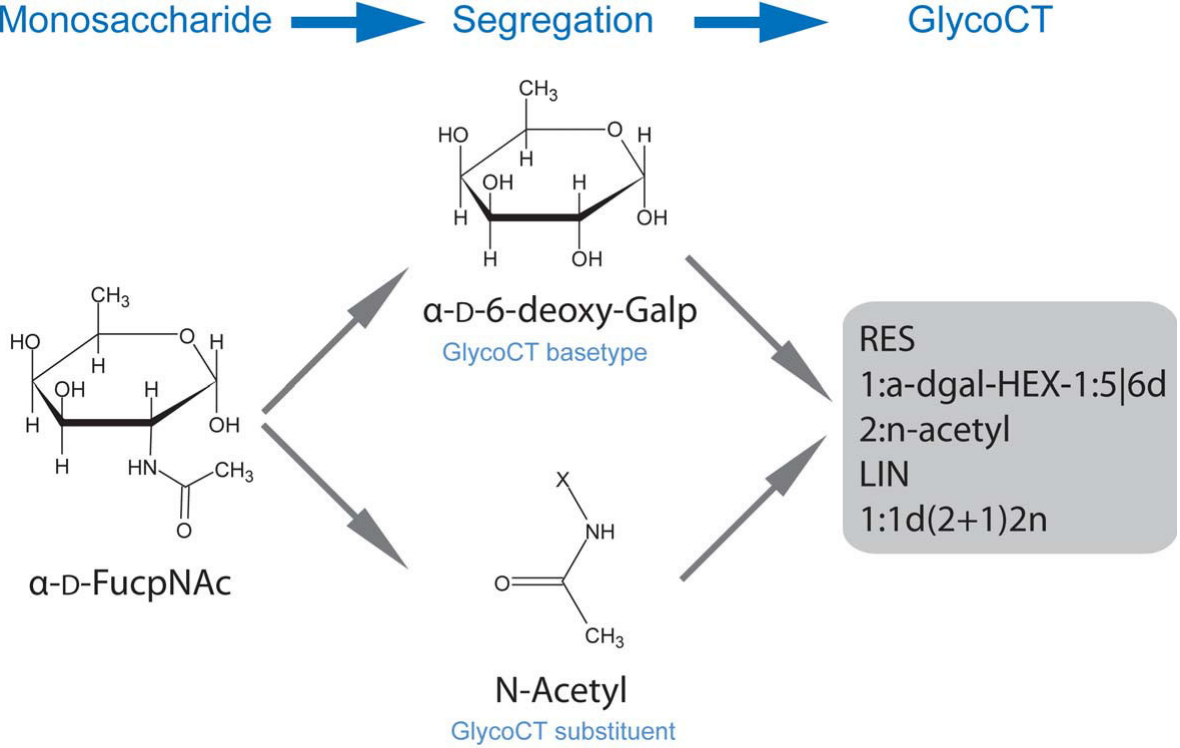
**Translation of residues to GlycoCT notation**. The example illustrates how the monosaccharide α-D-Fuc*p*NAc is separated into its GlycoCT basetype and substituent. The final GlycoCT representation of this residue is shown at the right.

We have extracted a total of 11599 residue names that occur in the existing databases and classified them into three sets. The first set consists of 5762 entities which encode *non-carbohydrate *chemical entities according to the GlycoCT definitions. These are mainly aglycons attached to the reducing end, such as amino acids, lipids or other small molecules. In some cases these entities can be attached to a terminal residue of a carbohydrate sequence. Non-carbohydrate entities are not processed further during the generation of the *GlycomeDB*, but their identity and connectivity are stored in the database for subsequent analysis. The second set of residues comprises 5180 monosaccharide names, which were successfully translated into the GlycoCT notation. During this process, the traditional monosaccharide name is divided into a *basetype *and *substituents *according to the GlycoCT definitions (see the example in Figure 3). This dictionary is used later for the translation process. The third set of residues comprises 657 names which cannot be interpreted or translated into GlycoCT notation. These are mostly invalid monosaccharide names and other unresolvable names (e.g. b-6daraHex3Me or <Man5-9>).

### Integration of taxonomic annotations

Taxonomic annotations for at least a subset of the records are available for GLYCOSCIENCES.de, BCSDB, CFG, CarbBank and GlycoBase (Lille); but, again, no common standard for information encoding is adhered to. GLYCOSCIENCES.de and BCSDB use the taxonomic resources (IDs) of the National Center for Biotechnology Information (NCBI) [24], while the others utilize free-text designators. Table 2 shows the total number of structures with taxonomic annotations and the number of species names or numerical IDs used in the various databases. It should be noted that not all assignments are species-specific; approximately 16.9% of the structures are simply associated with a genus, class, kingdom or superkingdom (e.g. eucaryota, bacteria, or fungi).

**Table 2:** Taxonomic annotations obtained from source databases

Database	Taxonomy data format	Structures with taxonomic annotation	Unique species names or IDs	Unique NCBI taxonomy IDs
BCSDB	NCBI ID	6747	451	451
CarbBank	free text	13521	2471	1594
CFG	free text	2966	273	240
GlycoBase (Lille)	free text	178	13	13
GLYCOSCIENCES.de	NCBI ID	5384	312	312

Five source databases provided taxonomic annotations in the format listed. For each database the numbers listed are: total structures with taxonomic annotations (column 3), unique species names or IDs found (column 4), and unique NCBI taxonomy IDs remaining after data integration and standardization in *GlycomeDB *(column 5). The large difference between the number of free-text names and assigned NCBI IDs is a result of the usage of different names for the same species and names which could not be translated to NCBI taxonomy IDs.

For *GlycomeDB *we have opted for the NCBI taxonomy. The free-text annotations used in other databases are mapped automatically to the NCBI taxonomy tree, and this mapping succeeded for 1896 datasets from a total of 2757 cases examined. Another 159 species names were resolved manually. The remaining 702 species names, mainly from CarbBank, could not be found in NCBI and were, therefore, not included in *GlycomeDB*. For each database the total number of species with a resolvable NCBI taxonomy ID are listed in Table 2. The mapping from text names to NCBI taxonomy IDs is stored in a local database and is used during the data integration process.

### JAVA application *GlycoUpdateDB*

**Figure 4:**
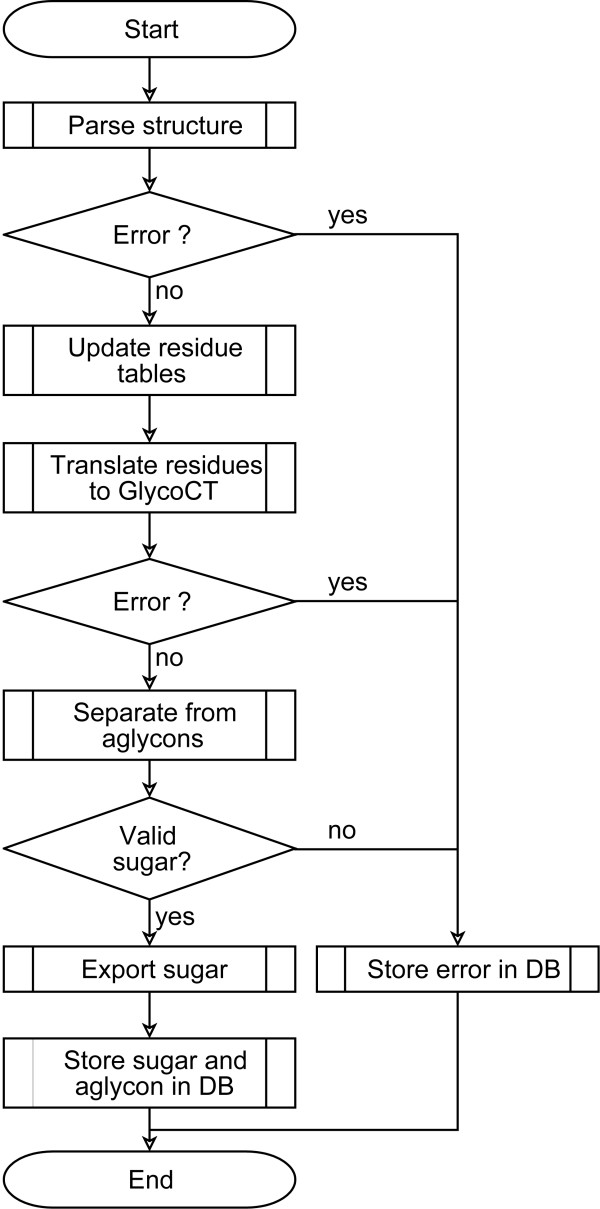
**Flow chart for structure translation**. The flow chart delineates how the carbohydrate structure translation process is applied for each sequence in its original encoding as retrieved from the source database. When no errors are detected, the result is a validated GlycoCT representation for the carbohydrate structure. Detected errors (grammatical, typographical) are stored separately and reported back to the curator of the source database.

*GlycoUpdateDB *is the application program which we have designed to carry out the integration of the interpretable data obtained from the resources described above. It is a JAVA application [25], depending on a PostgreSQL database [26], which can be configured by an XML file. The configuration file contains settings for the local database and instructions for the download and data integration process. Initially, database tables with dictionaries and mappings for the taxonomic data are required. The first stage of integration includes the download process with subsequent extraction of the data files to the local PostgreSQL database. *GlycoUpdateDB *supports the three download strategies shown in Table 1 and can also use locally resident files (e.g. static databases such as CarbBank). The second stage of integration involves the actual translation of all downloaded and interpretable structures into their corresponding GlycoCT representations and storing of the translated structures in *GlycomeDB*. Figure 4 shows the workflow applied for each structure.

For the EUROCarbDB project [27] we created an object model for carbohydrate sequences. This model has been implemented in JAVA and is also used during the sequence translation and data integration process. Each downloaded carbohydrate sequence is parsed, and a JAVA object for each structure is created. If a parsing error is detected, the process stops and the error is stored in the local database. Typically, such errors arise from typographical errors in the sequence notation, resulting in violations of the grammars. After the parsed sequence has been loaded into the object model, the residue names are translated to their GlycoCT equivalents. If a dictionary entry for a particular residue name is missing, then the residue name is recorded in the database for a later manual curation process. If all residue names are known and valid, then the structure is converted into the GlycoCT notation, otherwise the event is recorded in the local database as an error.

Most carbohydrate structure databases also store non-carbohydrate entities which are attached to the carbohydrate sequence. In some databases it is even possible to encode sequences consisting of more than one aglycon residue and several carbohydrate chains, e.g. highly glycosylated peptides. Since *GlycomeDB *is mainly focused on carbohydrate sequences, we have included a program module which separates aglycons from the carbohydrate chains and stores the aglycons separately in the database. After the formal quality check described below, the carbohydrate structure present in the object model is exported to GlycoCT_{condensed}_, GlycoCT_{XML} _and GlydeII, and stored in the structure tables of the local database, along with the access key (ID) used in the original database. The taxonomic annotations for each structure are also deposited in the local database using the corresponding numerical NCBI taxonomy ID.

### Database *GlycomeDB*

*GlycomeDB *consists of several database schemata with tables that store all downloaded and generated datasets. Table 3 shows an overview of all schemata, the number of tables in each schema and a description of the content. A more detailed description of the database tables and schemata can be found on the webpages of *GlycomeDB*: 

**Table 3:** Database schemata in GlycomeDB

Schema name	Tables	Description
core	16	Monosaccharide translation tables and integrated structure table
dictionaries	6	Dictionaries, mainly for species-to-NCBI mapping
ncbi	6	Download of the NCBI taxonomy database and derived tables
raw_databasename	1–3	Schemata which contain the downloaded data from the source defined by databasename
remote	11	Associated data: original source IDs, taxonomic annotations, aglycons

*GlycomeDB *schemata are listed in alphabetical order, for each schema the corresponding number of tables incorporated and a short description of their content is given.

**Figure 5:**
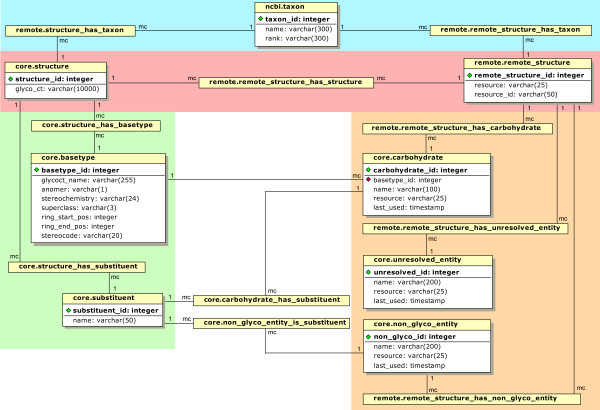
**Entity relationship diagram for *GlycomeDB***. This diagram represents some of the schemata, tables and connectivities incorporated in *GlycomeDB* (see text for details). The name at the top of each table has the format *schema_name.table_name*. All m-to-n tables are simply represented by this name within a yellow box; other tables are shown with a list of the important attributes. Primary keys of the tables are indicated with green markers while foreign keys have red markers. Labels describing the cardinalities of the relationships between tables are given in the modified Chen notation ("1" = one, "mc" = zero, one or many). The top section of the diagram illustrates the relationships between taxonomic annotations (blue background) and structures (red background). The original structures and the GlycoCT translation are linked to each other via the *remote.remote_structure_has_structure *table. The tables in the orange section represent the dictionaries for the residues used and their associations with the original structures. The green section includes the GlycoCT basetypes and substituents which have relationships with the GlycoCT-encoded structures.

Initially, the database contains the schemata *core *and *dictionaries*, with tables that include the dictionaries for residue translation and taxonomy mapping, and the schema *remote*, which has initially empty tables to be filled during data integration. During a *GlycoUpdateDB *run, a new schema is added for each downloaded database, following the naming convention *raw_databasename *(e.g. *raw_cfg*). These schemata contain the downloaded primary data from each of the external databases. Moreover, the schema *ncbi *is created and filled with a dump of the NCBI taxonomy database. The downloaded information in these schemata is used to fill the *remote *schema during the data integration process. Figure 5 shows various parts of the *GlycomeDB *database in an entity relationship diagram, with the taxonomic and structural parts at the top.

The red background in Figure 5 highlights the *structure *and *remote_structure *tables and their association. An entry in *remote.remote_structure *contains the origin or database name (*resource*) and the original ID (*resource_id*) of the structure. An entry in *core.structure *is a translated carbohydrate substructure of the original structure and consists of an ID and the unique GlycoCT encoding for this structure. The relationship between these two tables is an m-to-n (many-to-many) relationship, since a remote structure may encode more than one carbohydrate substructure (possible in CarbBank and GLYCOSCIENCES.de) and a given carbohydrate structure may be contained in several remote structure entries. Not shown in Figure 5 are the entities *core.structure_glycoct_xml *and *core.structure_glyde*, which contain each structure encoded in GlycoCT_{XML} _and GlydeII format, respectively.

The blue area at the top of Figure 5 shows the entities dealing with taxonomy. The table *ncbi.taxon *contains the NCBI taxonomy ID (*taxon_id*) and the scientific name and rank for each entry. Both *remote.remote_structure *and *core*.*structure *have m-to-n relationships to *ncbi.taxon*. For *remote.remote_structure *these relationships represent the taxonomic assignments from the original databases; for *core.structure *the relationships represent the assignments after data integration.

The bottom part of Figure 5 shows the tables of the database used for storing the compositions of the structures and for residue name translation. The relationship between remote structures and their residues is shown in the orange area. Each remote structure can have an m-to-n relationship to the three residue classes *carbohydrate*, *non_glyco_entity *(aglycon) and *unresolved_entity*. The residues in the table *core.carbohydrate *represent carbohydrates in the traditional perspective, i.e. a residue is a monosaccharide with a defined configuration and substitution (e.g. α-D-Glc*p*4Ac). Entries in *core.non_glyco_entity *are aglycons such as ceramide (Cer) or substituents that are not directly attached to the monosaccharides, since some sequence formats treat substitutents such as sulfates or phosphates as separate residues. Finally, *core.unresolved_entity *contains all newly appearing or unresolvable residues. The m-to-n relationships to the remote structure table can be regarded as composition tables, enumerating the usage of the residues. In the green section of Figure 5 the analogous dictionary and composition tables for the GlycoCT basetypes and substituents and their relationships to the GlycoCT structures are shown.

The relationships between the orange and the green sections are used for the translation of the remote residue namespace to the GlycoCT residue namespace. For example a carbohydrate corresponds to a basetype and may also contain several substituents. Therefore, each carbohydrate in *core.carbohydrate *has a *basetype_id *from *core.basetype *as a foreign key (red marker) and in addition, an m-to-n table to *core.substituent*. During carbohydrate translation each residue is looked up in these tables and translated accordingly to its GlycoCT representation. As described above, some of the non-glyco-entities correspond to substituents; therefore an m-to-n table between *core.non_glyco_entity *and *core.substituents *is used during the translation step.

## Utility and discussion

All numbers in the figures, tables and text relating to *GlycomeDB* are based on the version of *GlycomeDB* compiled in August 2008. This includes the numbers of residues, numbers of structures in the various source databases and *GlycomeDB* and the numbers of taxonomic annotations. Note that these numbers are subject to change as the database is periodically updated.

### Data quality

*GlycomeDB *is a database which integrates knowledge from other existing databases. Therefore, the quality of the data depends on the quality of the referenced databases and their curation processes. Most of the digitally available carbohydrate sequences stem from retrospective literature analysis, and in most cases errors can only be detected by re-examining the original publications, which is beyond the scope of this project. Nevertheless, we have added a validation module to *GlycoUpdateDB*, which checks each structure by validating the monosaccharide residues and linkages using formal criteria. This procedure is facilitated by the machine-readable monosaccharide notation of GlycoCT.

Linkages are checked against all possible substitution patterns for the monosaccharides involved, e.g. a fucose can not be the acceptor of a (1–6) linkage from another residue since a glycosidic linkage to the methyl group (C6) of fucose is not possible. Monosaccharides are confirmed to follow standard IUPAC naming conventions, e.g. 3-deoxy-*galacto*-Hexose is not a valid name, since the stereochemistry is overdetermined; the correct name is probably 3-deoxy-*xylo*-Hexose or possibly 3-deoxy-*galacto*-Heptose. Sequences containing errors will be automatically detected by the validation module and will *not *be integrated into *GlycomeDB*.

In addition to naming errors, we also found typographic errors in the sequence encoding (e.g. a bracket at a wrong position) which violate the established grammars or typographic errors in the residue names. All of these errors, which were automatically detected during data integration, were recorded separately in the database and were reported in manually generated reports to the responsible curators during multiple rounds. Many errors have been subsequently corrected, and we observe a steady increase in data quality for all databases concerned. However, the percentage of untranslatable sequences still remains relatively high for some sources (up to 11.5%). Further efforts in curation and software development are certainly needed.

### Utility

Through the integration of all public global resources, *GlycomeDB *has become the most comprehensive resource for carbohydrate structures worldwide and can be used by researchers to determine whether or not a given carbohydrate structure has been reported previously. The potential value of a unified carbohydrate sequence database for a wide range of applications (analysis, statistics, method development) is significant. Up to now, all analytical work in the glycosciences depended on single databases [28-30]. Annotation tools for experimental MS or NMR applications [31-33] use single databases for their structure prediction engines and could benefit from the broader information base provided by *GlycomeDB*.

### Results

The current downloadable version of *GlycomeDB *(August 2008) contains 33283 unique carbohydrate structures unambiguously defined by GlycoCT encoding and is updated on a weekly basis with the newest structures available. It should be noted that, according to our preliminary analyses, the total sequence space was artificially inflated due to assumptions made by the individual database initiatives. For example, some initiatives modified the original entries derived from CarbBank by changing a reduced monosaccharide (e.g. GlcNAc-ol) to its more probable naturally occurring state (Glc*p*NAc), while other databases retained the original CarbBank entry. This situation gives rise to two almost identical carbohydrate sequences as separate entries in *GlycomeDB*. For specific applications or analyses it may be desirable to exclude such multiple entries, but such a decision should not be part of the data integration process, which should leave the primary data intact.

**Figure 6:**
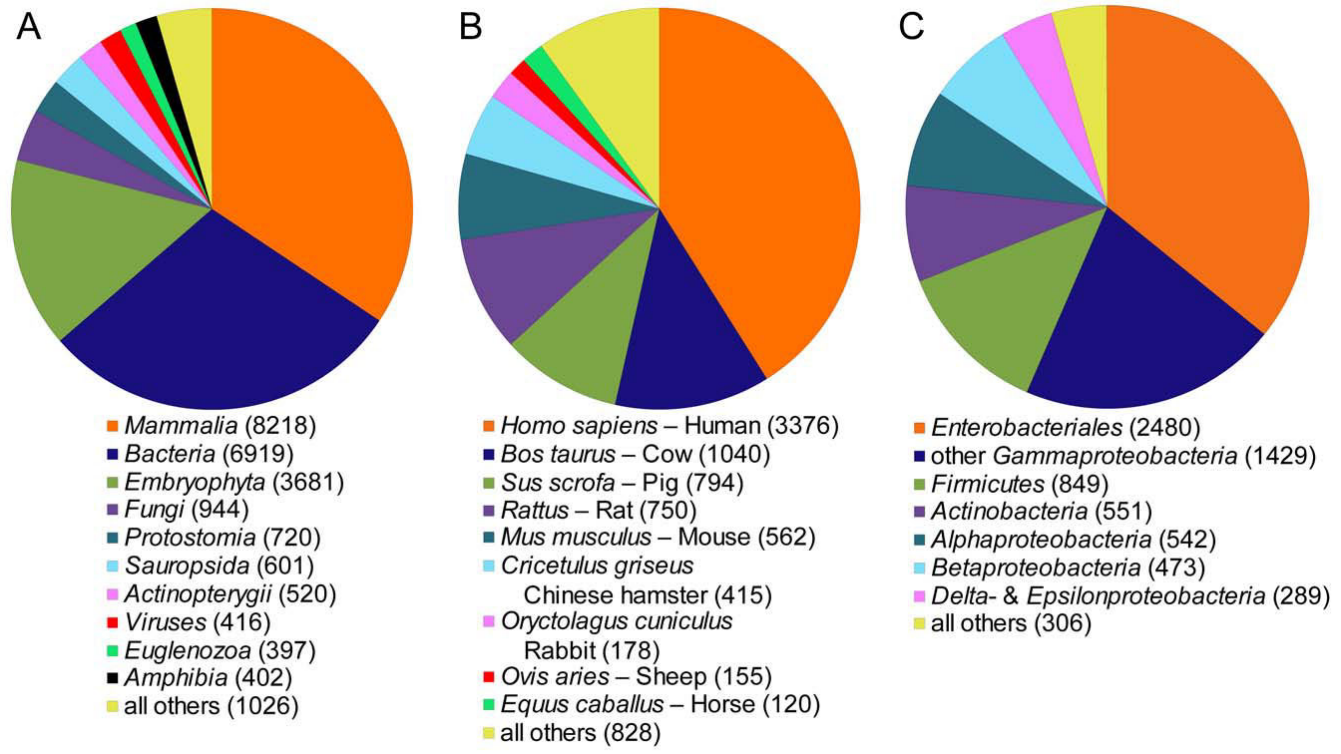
**Frequency distribution of carbohydrates in major taxonomic categories**. The pie charts show the frequency distribution (%) of taxon-structure tuples in *GlycomeDB *for **(A) **general taxonomic categories; **(B) **subclassifications within *Mammalia*, with common names added; **(C) **subclassifications within the domain *Bacteria*. The absolute number of occurrences for each taxonomic group listed in the legends is given in parentheses.

A total of 14535 structures were found to have at least one taxonomic annotation, and 1811 different taxons were referenced. A total of 23844 structure-taxon tuples are recorded in the database, and Figure 6 summarizes the numbers of entries assigned to various taxonomic groups and frequency distributions (pie charts) for (A) all major taxonomies, (B) *Mammalia *and (C) *Bacteria*.

*GlycomeDB *contains an index of all structures taken from the seven integrated databases and their taxonomic annotations. With this integrated database we are now able to find all structures that belong to a specific taxonomic group, e.g. all human carbohydrate structures. In addition, it is possible to query for the occurrence of a specified structure in each of the source databases and obtain a list of the corresponding source IDs for cross-linking to each of the original databases. Note that *GlycomeDB *does *not *include the entire contents of each source database (e.g. biological or biochemical information, literature references, etc) but does provide the necessary source IDs through which the user can access the original databases to obtain all information available for any carbohydrate structure contained in *GlycomeDB*.

### Outlook

The reduction of the error rate of *GlycomeDB *in collaboration with the individual database initiatives is an outgoing task. In the future, other databases which currently employ restricted access policies will probably move to the public domain (e.g. current discussions about GlycoSuiteDB). These databases will be integrated into *GlycomeDB *as soon as they become available. Currently, a web portal based on *GlycomeDB *is being developed to allow users to search the database. A prototype implementation has been completed, but at the time of this publication the structure search capabilities are still limited [34].

## Conclusion

We have created a new database, called *GlycomeDB*, which integrates the structural and taxonomic data of all major carbohydrate databases available in the public domain (BCSDB, GLYCOSCIENCES.de, CFG, KEGG, GlycoBase (Dublin), GlycoBase (Lille) and CarbBank). *GlycomeDB *is now the most comprehensive source for carbohydrate structures worldwide, and it will be updated at weekly intervals with the newest structures available from the source databases. The current *GlycomeDB *database contents and the application *GlycoUpdateDB *for the local installation and updating of *GlycomeDB *are now available via download and can be utilized by interested scientists.

The need for database development in glycomics has been emphasized frequently: "We need to be able to search databases for what is out there. Imagine genomics and proteomics without GenBank" (Ajit Varki) [35]. With this project we hope to provide a major step forward in the development of standardized, open-access databases for carbohydrate structures and related information pertinent to applications in the glycosciences.

## Availability and requirements

Three different mechanisms can be accessed via the web portal [34] for the distribution of the *GlycomeDB *contents and the application *GlycoUpdateDB*. Detailed installation instructions can be found on the download web pages .

### Download of structure data files

A compressed zip archive (3.3 MB) is available, containing all structures that have been integrated into *GlycomeDB*. The structures are stored in regular XML files according to the GlydeII specification and can be used by any software which supports this format. With these data the user is totally independent of our database and our internal structure encoding (GlycoCT).

### Download of an SQL dump

The second download possibility is a PostgreSQL dump (54 MB), which contains the complete *GlycomeDB* including all schemata and tables. This dump can be imported to local PostgreSQL installations.

### Distribution of installation routines

Finally, the JAVA application *GlycoUpdateDB *and a core database dump with dictionaries are available for download (2.5 MB). After installation of the core database, the local database can be filled using *GlycoUpdateDB*. A local PostgreSQL database and JAVA JRE 1.5 are required. Thus, the user can update the local installation of *GlycomeDB *at any time to obtain the newest structures available from the original source databases.

For example, *GlycoUpdateDB *needs about 5 hours to generate the complete *GlycomeDB *database on a computer with a 3 GHz Intel Pentium 4 processor, 1 GB RAM and Internet access. The procedure involves the downloading of approximately 50 MB of data which are temporarily stored in the local file system. Operations on the NCBI taxonomy tree require about 100 min. Finally, about 3 hours are needed for the data integration stage.

## Authors' contributions

RR and SH designed the database and software and drafted the manuscript. RR was responsible for the parsers and data integration routines, while SH created the data acquisition routines and handled the curation of the monosaccharide dictionaries and the taxonomic annotations. CWL and TW provided guidance for the project and helped with the connections to the other database initiatives.

## Supplementary Material

Additional file 1 EBNF definitions for carbohydrate sequence encoding schemata. Click here for file
