# Supplementary material for: GlycomeDB – integration of open-access carbohydrate structure databases
Source: BMC Bioinformatics. 2008 Sep 19;9:384. doi: 10.1186/1471-2105-9-384 (PMC2567997; doi:10.1186/1471-2105-9-384)
Supplement: Additional file 1 — EBNF definitions for carbohydrate sequence encoding schemata. [file 1471-2105-9-384-S1.doc]

This document contains the extended Backus–Naur form (EBNF) for the carbohydrate sequence formats used in the various source databases.

**Common production rules**

The rules in this section are common production rules which are used in all grammars.

number = "0" | ( "1" | ... | "9" ) { "0" | "1" | ... | "9" } ;

character = "a" ... "z" | "A" ... "Z" ;

**LINUCS**

start = "[" "]" "[" residuename "]" "{" { subresidue } "}"

| "[" "]" "[" "L" "I" "N" "K" "]" "{" "[" "(" "U" "N" "T" "I" "L" "+" linkage ")" "]" "[" residuename "]" "{" { subresidue } "}" "}" ;

linkage = ( number { "/" number } | "?" ) [ ">" [ repeatcount ] ] ;

residuename = symbol { symbol } ;

symbol = character | "0" | ... | "9" | "(" | ")" | ";" | "," | ":" | ">" | "<" | " " | "\" | "'" | "-" | "?" | "_" | "+" | "/" | "." | "=" ;

subresidue = "[" "(" link_r + link_n ")" "]" "[" residuename "]" "{" { subresidue } "}" ;

link_n = ( number { "/" number } [ ">" [ repeatcount ] ] ) | "REPEAT" | "?" | "CYCLIC" | "N" | "S" | "P" ;

repeatcount = character [ "X" ] | number [ "X" ] ;

link_r = number { "/" number } | "?" | "<" number { "/" number } | "<" "?" | "N" | "S" | "P" ;

**Oxford notation**

start = corefucose [ "X" ] ( m | a ) ;

corefucose = [ "F" "(" "3" ")" ] [ "F" "(" "6" ")" ] ;

m = "M" m_type [ "B" ] ;

m_type = "1" | "2" d | "3" | "4" d | "5" | "6" d | "7" d | "8" d | "9" ;

d = [ "D" number { "," "D" number } ] ;

a = "A" number "[" number { "," number } "]" [ "B" ] [ "F" [ "(" number { "," number } ")" ] number ] [ g ] ;

g = "G" [ "(" number { "," number } ")" ] number [ "F" [ "(" number { "," number } ")" ] number ] branch_rest;

branch_rest = ["G" "a" [ "(" number { "," number } ")" ] number ] ["S" [ "(" number { "," number } ")" ] number ];

**KCF**

start = head nodes edges [ bracket ] "/" "/" "/" [ "\n" ] ;

head = "E" "N" "T" "R" "Y" " " { " " } go_number " " { " " } "G" "l" "y" "c" "a" "n" "\n" ;

go_number = "G" ( "0" | ... | "9" ) { ( "0" | ... | "9" ) } ;

nodes = "N" "O" "D" "E" " " { " " } number "\n" node { node } ;

node = { " " } number " " { " " } glycan_name " " { " " } signed_dec_number " " { " " } signed_dec_number "\n" ;

glycan_name = symbol { symbol } ;

edges = "E" "D" "G" "E" " " { " " } number "\n" { edge } ;

edge = { " " } number " " { " " } number [ ":" link_information ] " " { " " } number [ ":" link_information ] "\n" ;

link_information = [ "a" | "b" ] [ ( number [ "," number ] ) | ( * [ "1" ] ) ] ;

symbol = character | "0" | ... | "9" ;

bracket = "B" "R" "A" "C" "K" "E" "T" bracket_line bracket_line bracket_final ;

bracket_line = { " " } number " " { " " } signed_dec_number " " { " " } signed_dec_number " " { " " } signed_dec_number " " { " " } signed_dec_number "\n" ;

bracket_final = { " " } number " " { " " } ( signed_dec_number " " { " " } signed_dec_number " " { " " } ( "n" [ "-" number ] | number [ "-" number ] ) | "n" | "m" ) "\n" ;

signed_dec_number = [ "-" | "+" ] number [ "." ( 0 | ... | 9 ) { 0 | ... | 9 } ] ;

**BCSDB**

start = "-" [ "P" "-" ] linkage_rest ")" rsugarchain "(" linkage_rest "-" [ "P" "-" ]

| "P" "-" ")" [ sidechain ] sugarchain

| sugarchain ;

rsugarchain = [ sidechain ] residue { linkages [ sidechain ] residue } ;

sugarchain = residue { linkage [ sidechain ] residue } [ "(" linkage_rest "-" residue ] ;

sidechain = "[" [ ":" ] side_residue [ sidechain_follow ] "]" ;

side_residue = residue linkage | "P" "-" linkage_rest ")" | "S" "-" linkage_rest ")" ;

sidechain_follow = "," side_residue [ sidechain_follow ]

| sidechain residue linkage [ sidechain_follow ]

| residue linkage [ sidechain_follow ]

| ":" side_residue [ sidechain_follow ] ;

residue = ( character | "?" ) { character | number | "?" | "-" } ;

linkage = "(" linkage_rest "-" { "P" "-" } linkage_rest ")" ;

linkage_rest = "?" | number ;

**GlycoBase(Lille) notation**

Each residue line in the GlycoBase(Lille) notation is parsed separately.

start = "[" "i" "n" "c" "r" "." [ " " ] number { "," number } "]" " " residue_name " " linkage " " "(" symbol { symbol } ")" " " enhancement ;

residue_name = "&" ( ( "b" "e" "t" "a" ) | ( "a" "l" "p" "h" "a" ) ) ";" symbol { symbol } "<" "i" ">" character "<" "/" "i" ">" ;

linkage = "(" number "-" number ")" ;

enhancement = symbol { symbol | "(" | ")" | number | " " } ;

symbol = character | "-";

**GlycoMinds encoding**

Underdetermined terminal residues are separated from the main sequence and are parsed by the subbranch production rule. The main sequence is then parsed with the start rule.

start = residuename { position { “(“ subbranch “)” } residuename } ;

subbranch = residuename position { { “(“ subbranch “)” } residuename position } ;

position = "?" | number { "/" number } ;

residuename = ( "?" [ modification ] ( "?" | "a" | "b" | "o" ) )

| ( symbol { symbol } [ modification ] ( "?" | "a" | "b" | "o" ) ) ;

symbol = character | "’" | "^" | "~" ;

modification = "[" ( character | number ) { ( character | number ) } "]" ;
